# Seabirds Enhance Primary Producer and Consumer Isotope Signals on a Sub‐Tropical Island

**DOI:** 10.1002/ece3.71636

**Published:** 2025-07-27

**Authors:** Megan L. Grant, Suzie M. Reichman, Alexander L. Bond, Jennifer L. Lavers

**Affiliations:** ^1^ Instititute for Marine and Antarctic Studies University of Tasmania Newnham Lutruwita/Tasmania Australia; ^2^ Adrift Lab Underwood Lutruwita/Tasmania Australia; ^3^ School of BioSciences University of Melbourne Parkville Victoria Australia; ^4^ Bird Group The Natural History Museum Hertfordshire UK; ^5^ Gulbali Institute Charles Sturt University Wagga Wagga New South Wales Australia

## Abstract

Seabirds are long‐range transporters of nutrients, linking their marine feeding grounds with their terrestrial breeding and roosting sites. Seabirds can influence the terrestrial environment in which they reside by depositing nutrient‐rich guano, which acts as a natural fertiliser. Here, we determined the nutrient content of Sable Shearwater (*Ardenna carneipes*) guano and used stable isotope analysis to determine changes in isotope signals and nutrient concentrations through the terrestrial environment across three sites on Lord Howe Island, Australia: active shearwater colony, abandoned shearwater colony and un‐colonised area. The concentration of nitrogen in Sable Shearwater guano was like other Procellariiformes, and this was reflected in the palm leaves and invertebrates (slugs) sampled from seabird colonies, which were enriched in δ15N relative to the control site. In contrast, nitrogen stable isotope values in soils were not significantly different among sites, indicating the presence of historic seabird colonies. Guano was rich in phosphorous and potassium, which fertilised soils in the shearwater colony compared to sites without seabirds present. It is expected that the island will experience a reduction in nutrient inputs from guano because the shearwater population is in decline, and this may result in changes to vegetation assemblages in the colonies over time.

## Graphical abstract

Many seabird populations are decline, this means fewer marine‐derived nutrients are being deposited on islands. On Lord Howe Island, soil collected from active shearwater colonies contained elevated concentrations of nitrogen and phosphorus compared to abandoned colonies. The loss of birds, and nutrients, may result in changes to vegetation assemblages over time.

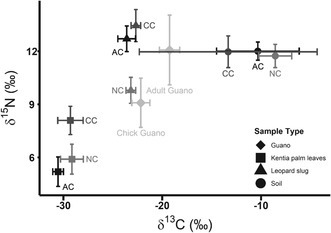

## 1 Introduction

Birds are one of the most diverse groups of ecosystem service providers and exhibit an extensive range of ecological functions (Whelan et al. 2008). The United Nations Millennium Ecosystem Assessment identified four principal types of ecosystem services—namely cultural, provisioning, regulating and supporting—and birds contribute to all four (Michel et al. 2020; Millennium Ecosystem Assessment 2005). For example, culturally, birds generate significant public interest (Belaire et al. 2015); provisioning services such as food and materials (e.g., feathers) are provided by domesticated and wild birds (Mosbech et al. 2018); birds regulate their landscape through biogeomorphological changes driven by guano‐fostered vegetation growth (Reijers et al. 2024), seed dispersal, pollination and pest control (Şekercioğlu 2006); and birds can support ecosystems through ecosystem engineering (e.g., burrowing), and vectoring (i.e., transporting) and deposition of nutrients (Bancroft, Garkaklis, and Roberts 2005; Grant et al. 2022).

While terrestrial birds can transport existing nutrients within an island, seabirds are more commonly recognised for their role in transferring nutrient subsidies between marine and terrestrial ecosystems. Seabirds link marine and terrestrial environments by depositing marine‐derived nutrients at breeding and roosting grounds through guano and other allochthonous inputs (e.g., feathers, carcasses; Zwolicki et al. 2015). The nutrients found in guano are predominately nitrogen and phosphorus, but also include potassium, calcium and other macro‐ and micronutrients, many of which are essential to maintaining ecosystem health and integrity (De La Peña‐Lastra 2021; Otero et al. 2018). Seabird colonies can be influential irrespective of location; however, colonies on remote islands are often larger (due to the absence of predators) and more beneficial for the recipient environment than those based on mainlands (Caut et al. 2012; Jones 2010). While mainlands have a diverse and substantial array of nutrient inputs (e.g., agricultural runoff, industrial effluent and emissions; Nedwell et al. 2002; Zhang et al. 2022), island ecosystems are generally isolated and have fewer sources of nutrient inputs in comparison (Benkwitt et al. 2022). Thus, islands that have populations of breeding and roosting seabirds gain substantial beneficial nutrients from their guano (typically around 65,000 kg of nitrogen and 11,000 kg of phosphorus per island per year; Otero et al. 2018; Steibl et al. 2024). Thus, nutrient subsidies provided by seabirds are often the predominant external source (Anderson and Polis 1999; Buelow et al. 2018), with many islands highly dependent on seabirds for sustaining their ecosystems (Otero et al. 2018; Steibl et al. 2024).

The effects of guano deposition are many which in turn increases primary productivity (Caut et al. 2012). The presence of seabird colonies has led to increases in vegetation cover (Bukancinski et al. 1994), shifts in species compositions (Wait et al. 2005), increases in invertebrate abundance (Fukami et al. 2006; Polis and Hurd 1996), changes to populations of mammalian and reptilian scavengers and predators (Barrett et al. 2005; Markwell and Daugherty 2002; Richardson et al. 2019) with guano also playing a key role in moderating climate (Boyer et al. 2025). Thus, when guano is deposited in colonies it acts as a natural fertiliser for the soil and surrounding environment (Jones 2010; Kolb et al. 2010). Consequently, seabird colonies are often hotspots of productivity, and the presence of seabirds can result in marked and visible differences in ecosystems compared to areas without seabirds (Payne and Moore 2006; Young et al. 2011).

Globally, seabird populations are in decline due to a range of pressures, such as climate change and fisheries bycatch (Dias et al. 2019), and are among the fastest declining bird groups (Croxall et al. 2012). Fewer seabirds will result in lower guano deposition and consequently a decline in nutrient input in the vicinity of seabird colonies. When nutrient subsidies are reduced, islands and the surrounding marine environment can be negatively impacted through reductions in soil quality, which have far‐reaching impacts for fauna and flora (Croll et al. 2005; Fukami et al. 2006). Thus, reductions in seabird populations that inhabit islands with few alternative nutrient inputs could potentially be damaging to recipient ecosystems (Graham et al. 2018; Maron et al. 2006).

The world's largest Sable Shearwater (formerly Flesh‐footed Shearwater; *Ardenna carneipes*) (Bond and Lavers 2024) colony is on Lord Howe Island, Australia, in the Tasman Sea (Lavers et al. 2019). The species breeds annually between September and May, during which time large amounts of guano are deposited on the island (Table S1; Reid et al. 2013). There is a visually apparent relationship between the shearwaters and the vegetation within the sub‐colonies on Lord Howe Island; where the shearwaters burrow, kentia palms ( *Howea forsteriana* ) thrive but few other plant species exist, and when the burrows stop, the rainforest becomes more diverse (authors' pers. obs.). Seabird‐vegetation relationships like these are not uncommon, with seabird dominated environments often hosting unique plant and invertebrate assemblages (Ellis 2005; Young et al. 2011). However, there is concern that this distinctive forest structure may change over time as the shearwater population has experienced a significant decrease over recent decades (Lavers et al. 2019), and concomitantly the colonies on Lord Howe Island have seen a decline in guano and associated nutrients as well as increased exposure to chemical pollutants (Grant et al. 2024).

The objectives of this study were to explore the intimate relationship between the Sable Shearwaters of Lord Howe Island and the broader ecosystem by analysing adult and chick guano for macronutrients (N, P, K) and to determine the isotopic signature of guano‐associated nutrients through soil, kentia palms and soil invertebrates (slugs). The influence of the shearwaters on their terrestrial environments was assessed by comparing the nutrient concentration of samples from three sites; an active shearwater colony (‘treatment site’), abandoned shearwater colony and an un‐colonised area (‘control site’). Nutrient signatures were examined using stable carbon and nitrogen isotopes which can be useful for tracing marine‐derived nutrients because of the distinction in marine *δ* ^15^N and *δ* ^13^C compared to terrestrial N and C stable isotopes (Harding et al. 2004). We hypothesised that the soil, kentia palms and soil invertebrates (slugs) from the active shearwater colony would be enriched in ^15^N relative to two additional sampling sites (abandoned colony, un‐colonised area), and this would inform us on the linkages between the shearwaters and the Lord Howe Island ecosystem, and potential changes to the islands' ecology that may take place if the shearwater population continues to decline (Lavers et al. 2019).

## 2 Methods

### 2.1 Study Area

**FIGURE 1:**
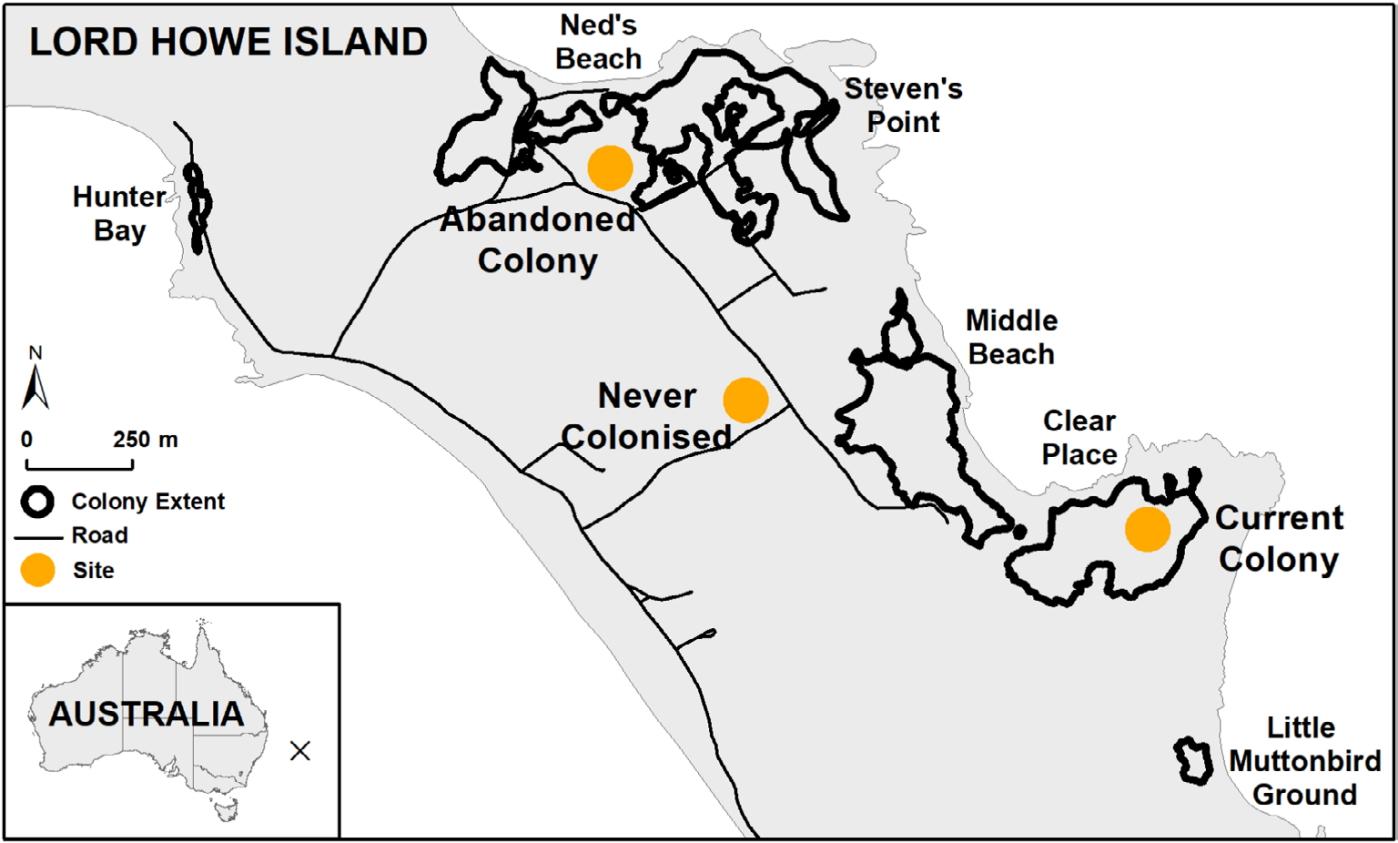
Map of the six Sable Shearwater (*Ardenna carneipes*) colonies on Lord Howe Island (Hunter Bay, Ned's Beach, Steven's Point, Middle Beach, Clear Place, Little Muttonbird Ground). Colony extents, as of 2018 (Lavers et al. 2019), are outlined in black. The three sampling sites (an active shearwater colony, ‘current colony’, an abandoned colony, ‘abandoned colony’ and an area that has never hosted a shearwater colony, ‘never colonised’) are indicated by orange circles. The location of Lord Howe Island is indicated by a black ‘X’ in the inset map.

The study was carried out on Lord Howe Island (1455 ha, 31°33′ S, 159°05′ E), located approximately 600 km east of Australia in the Tasman Sea (Figure 1). The island was formed 6.3–7 million years ago from volcanic activity and is home to a diverse array of endemic flora and fauna (Harris et al. 2005). In 1982, the whole island was added to the UNESCO World Heritage List and 75% of the island is a permanent preserve (Hutton et al. 2007). The climate is classified as subtropical with an annual mean temperature of 19.2°C (range = 14°C–25°C) and an annual rainfall of 1645 mm in the lowlands; however, rainfall in the southern mountain region is higher (Auld et al. 2010).

Discovered by the British Navy in 1788, Lord Howe Island is one of the last places to be settled by humans, with a small, permanent population established in 1834 (Anderson 2003). While some areas were cleared of native vegetation, and around 15% remains cleared to this day, 75% of the island is protected as a Permanent Preserve, and the whole island was declared a UNESCO World Heritage Site in 1982 (Hutton et al. 2007). While a number of invasive mammals have been introduced to the island, most have been eradicated (cats, mice, rats; Harper et al. 2020) and there is minimal evidence that rodents impacted Sable Shearwater chicks or adults (authors pers. obs.). However, early settlers often harvested seabirds and their eggs, with Sable Shearwaters being targeted for seasonal cultural events (Carlile 2003).

Sable Shearwaters breed in six areas on the northern half of the island (Clear Place, Little Muttonbird Ground, Middle Beach, Ned's Beach, Hunter Bay, Steven's Point; Figure 1). These colonies are characterised by lowland coastal rainforest dominated by kentia palms and few other plant species (e.g., Lord Howe Island banyan; *Ficus macrophylla* subsp. *columnaris*) (Sheringham et al. 2016), and are on sandy soils (Figure S1). For this study, we selected the largest colony, Clear Place, as our ‘treatment’ site. We then sampled two other sites. We selected a site that was previously occupied by an active Sable Shearwater colony but was abandoned in the last 20 years (Lavers et al. 2019), and an area with a similar forest structure but an absence of Sable Shearwaters (and other seabirds) as our control site (Table S2). The control site has never hosted an active seabird colony in recorded history (i.e., since Lord Howe Island was colonised by humans in 1833) (Paramonov 1960) but it is possible that there were seabirds at this site prior to human habitation. For the purposes of our study, it is the most suitable site available on the island to use as a control. Both the control site and the abandoned site have similar soil to the active colony (Figure S1), and while they also have kentia palms, there is a much greater variety of plant species present. The vegetation structure of these two sites is ‘mid‐high to very tall, closed forest’ and have a mixed canopy of cottonwood (*Celtis conferta* subsp. *amblyphylla*), maulwood (*Olea paniculate*), blackbutt (*Cryptocarya triplinervis* var. *triplinervis*), greybark (*Drypetes deplanchei*), kentia palm and banyan, as well as vines (variety of species including *Parsonia howeana* and *Smilax australis*) and a sparse ground cover (ferns, *Asplenium milnei*; sedges, *Carex brunnea* ; perennials, *Oplismenus imbecillis*) (Sheringham et al. 2016). The three study sites were > 600 m apart.

### 2.2 Sample Collection and Approvals

All applicable institutional and national guidelines for the care and use of animals were followed. Samples were obtained with permission from the Lord Howe Island Board (permit no. LHIB 07/18), the University of Tasmania Animal Ethics Committee (permit no. A0017677 and A0018480), and the New South Wales Department of Environment and Heritage (licence no. SL102382 and SL100169).

Most sampling was conducted in April and May 2019 (*n* = 43 samples) which coincided with the end of the Sable Shearwater breeding season. A small number of adult guano samples (*n* = 16) were also collected in October 2020 (during the COVID‐19 pandemic) which overlapped the beginning of the breeding season, and additional chick guano samples were collected in April/May 2021 (*n* = 10; chick samples were too small to be analysed individually, so were pooled into three combined samples). From each of the three study sites, we sampled kentia palm fronds, soil and soil invertebrates (leopard slugs *Limax maximus* ). Five quadrats (3 × 3 m) at least 10 m apart were randomly selected within each site, and the locations were recorded with a handheld GPS (±3 m; Table S2).

Soil samples were collected from a single, randomly selected point within each quadrat. Prior to digging, all leaf litter was removed from the surface to ensure a clean sample. Approximately 500 g of soil was collected using a clean plastic trowel from 0 to 5 cm and 25 to 30 cm depths and placed into separate snap‐lock bags. These depths were selected as they represent soil that is in contact with shearwaters and their guano (Powell et al. 2007). The top layer (0–5 cm) receives guano directly when shearwaters defecate on the colony surface. As Sable Shearwater burrows can be up to 3 m long (Dyer 2001), it is not possible to collect soil samples directly from burrows; thus, the 25–30 cm soil samples were used as a proxy. Soil samples were weighed to the nearest 0.1 g before being air dried to a constant mass. A total of 30 soil samples were collected (two depths × five quadrats × three sites).

The youngest fully expanded leaf from a kentia palm frond was collected from one kentia palm per quadrat. Kentia palms were the most abundant primary producer at all sites. Palm leaves were weighed to the nearest 0.1 g before being oven dried at 60°C until a constant mass was achieved (minimum of 24 h). A total of five leaves per site were collected.

Introduced leopard slugs are primary consumers and ubiquitous across the lowland of Lord Howe Island. Individuals were opportunistically spotted at night with a spotlight. Slugs were left overnight to empty their bowels before being killed by deep freezing. Slugs were then placed into an oven at 60°C for a minimum of 24 h or until a constant mass was achieved. All samples (slugs, leaves, soil) were kept at an ambient temperature in a dry, dark place until analysis.

Fresh shearwater guano (< 2 h old) was opportunistically collected at night from within the Ned's Beach colony (Figure 1) when guano was found on a suitable surface for removal (i.e., leaf, clothing, tarpaulin). All other fresh guano was collected by placing a shearwater (adult or 90‐day old fledgling) in a plastic lined box for a < 10 min. Birds were removed as soon as guano was defecated or after 10 min if no defecation occurred. Guano was scraped into an Eppendorf tube using a clean metal spatula, labelled and frozen at −20°C until analysis. In 2019, 22 adult and eight chick guano samples were collected, and these were used for stable isotope analysis. In October 2020, 16 adult guano samples were collected and due to the small sample volume, samples were pooled into five samples. In April/May 2021, 10 chick guano samples were collected and pooled into three samples for the same reason. The 2020 and 2021 samples were analysed by inductively coupled mass spectrometry (ICP‐MS).

### 2.3 Sample Analysis

Guano, leopard slugs and kentia palm leaf samples were freeze‐dried for 48–72 h to ensure they were completely moisture‐free before homogenization with a mortar and pestle (guano and slugs) or hammer mill (leaves) into a fine powder.

Stable isotope analysis and ICP‐MS were conducted at the Environmental Analysis Laboratory at Southern Cross University (Lismore, NSW, Australia). Total N (%), *δ* ^15^N and *δ* ^13^C for all samples (soil, guano, leopard slugs and leaves) were analysed on a Thermo Fisher Delta V plus isotope ratio mass spectrometer (IRMS). The IRMS was coupled to a Thermo Fisher Flash elemental analyser via an interface (Thermo Fisher Conflo IV). The isotope ratios were calculated as a permil (‰) deviation from atmospheric N (*δ* ^15^N) and the international limestone standard Vienna PeeDee Belemnite (VPDB; *δ* ^13^C):

$$\delta \left(‰\right)=\frac{\left(R_{\text{sample}}-R_{\text{standard}}\right)}{R_{\text{standard}}}$$

where *R*_sample_ is the ratio of the heavy to the light isotope and *R*_standard_ is the corresponding ratio for the standard. Samples were measured along with working standards (Glycine, Glucose, Collagen) to ensure measurement accuracy, and these standards had previously been calibrated against international reference materials (USGS64, USGS65; Table S3).

All samples were analysed by ICP‐MS to determine concentrations of P and K. Approximately 0.4 g aliquots of soil samples were digested on a hot block digestor for 1 h at 120°C in aqua regia (7.5 mL HCl:2.5 mL HNO_3_; RCI Labscan brand trace metal grade). Two blanks and a standard reference material (SRM; Sandy loam 2 CRM020) were digested with the soil samples, and duplicates were carried out every tenth sample. For the remaining sample types (guano, kentia palm leaves, leopard slugs), approximately 0.4 g of every sample were digested with 10 mL of 70% HNO_3_ (RCI Labscan brand trace metal grade) on a heat block at 120°C for 1 h. Two blanks and two SRMs (Dogfish liver DOLT‐5, Peach leaves NIST‐1547) were digested in the same manner as biological samples, and duplicates were carried out every tenth sample. Sample volumes for guano, leaves and slugs were small; therefore, there was insufficient sample mass to analyse any in duplicate. After all samples (soil, guano, leaves and slugs) were digested, all were made up to a final volume of 25 mL using ultrapure water (> 18.2 MΩ cm) and then diluted 10× (1 mL sample:9 mL ultrapure water). Recoveries of the SRM are presented in Table S3.

A Perkin Elmer NexION 2000B ICP‐MS (1600 W, 0.9 L/min nebuliser gas flow, 15 L/min plasma gas flow) was used to analyse P and K all samples. Kinetic Energy Discrimination mode, using helium (99.9995% purity) as the collision gas (4 mL/min), was used with some elements to reduce molecular interferences. Samples analysed by ICP‐MS are reported as percentage (%) dry weight (dw).

### 2.4 Statistical Analysis

All statistical analyses were completed in R 4.2.2 (R Core Team 2022). When nutrient concentrations were below the limit of quantification (LOQ), a number equal to 0.5 × LOQ was used to replace the missing value (Mikkonen et al. 2018). General linear models were used to test for differences between shallow and deep soil samples across sites. No significant differences were observed for all nutrient types (with the exception of *δ* ^15^N), thus soil depth was not included as a relevant factor in statistical analyses (i.e., each soil sample, regardless of depth, was treated as a replicate, with 10 replicates per site). A principal component analysis (PCA) was used to visualise relationships among parameters (e.g., N, P) measured in soil, vegetation and invertebrate samples from each site to see if variation could be partitioned to sites.

Residuals were checked for normality using Shapiro–Wilk significance tests and for skewness using D'Agostino tests. For normally distributed data, general linear models were used to test for significant differences in nutrient concentrations in sample types (soil, kentia leaves, leopard slugs) across our three sampling sites (current colony, abandoned colony, uncolonised site). Tukey's post hoc tests were then used for pairwise comparisons. For all other data, non‐parametric Kruskal–Wallis tests with post hoc Bonferroni‐corrected Dunn tests were used. Differences were considered significant when *p* < 0.05. Data are reported as mean ± standard deviation (SD).

## 3 Results

### 3.1 Nutrients and Stable Isotopes in Guano

Sable Shearwater guano was not significantly different between adults and chicks for most nutrients and stable isotopes, with some exceptions (Table 1). Adult *δ* ^13^C (−19.2‰ ± 1.0‰) and *δ* ^15^N (12.1‰ ± 2.0‰) values were significantly greater than the corresponding values in chicks (*δ* ^13^C: −22.2‰ ± 0.9‰; *F*_1,28_ = 49.26, *p* < 0.001; *δ* ^15^N: 9.1‰ ± 1.4‰; *F*_1,28_ = 27.07, *p* < 0.001; Table 1).

**TABLE 1:** Sable Shearwater (*Ardenna carneipes*) guano from adults and chicks on Lord Howe Island.

Variable	Adult	Chick	*H*/F	df	*p* value
N	20.81 ± 5.65^a^	19.64 ± 4.67^a^	0.56*	1	0.453
P	1.86 ± 1.11^a^	1.02 ± 0.16^a^	2.87	1,6	0.097
K	1.57 ± 0.64^a^	2.05 ± 0.63^a^	3.71	1,6	0.103
*δ* ^13^C	−19.2 ± 1.0^b^	−22.2 ± 0.9^a^	49.26	1,28	< 0.001
*δ* ^15^N	12.1 ± 2.0^b^	9.1 ± 1.4^a^	15.36	1,28	0.001

*Note:* Values are mean ± standard deviation (SD). General linear models (test statistic = *F*) were used for all variables, except for %N which were compared with non‐parametric Kruskal–Wallis tests (test statistic = *H*; denoted by asterisk [*]). Significant differences between adult and chick guano samples are indicated by different superscript letters. *p* values are shown in full unless *p* < 0.001. Total N, P and K were measured as percentage (%) and *δ* ^15^N and *δ* ^13^C as permil (‰).

### 3.2 Nutrients and Stable Isotopes in Soil

**FIGURE 2:**
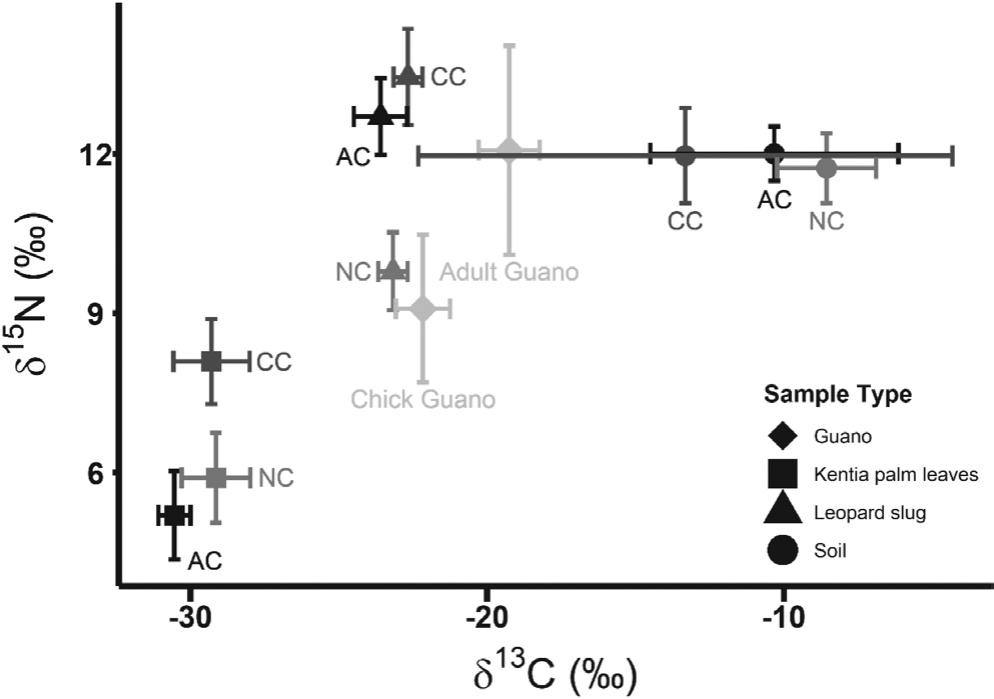
The mean (±SD) stable carbon (*δ* ^13^C ‰) and nitrogen (*δ* ^15^N ‰) isotope values from Sable Shearwater adult and chick guano, kentia palm leaves, leopard slugs and soil samples collected from three areas on Lord Howe Island, Australia: a current shearwater colony (CC), an abandoned shearwater colony (AC) and an area that has never been colonised by shearwaters (NC).

**FIGURE 3:**
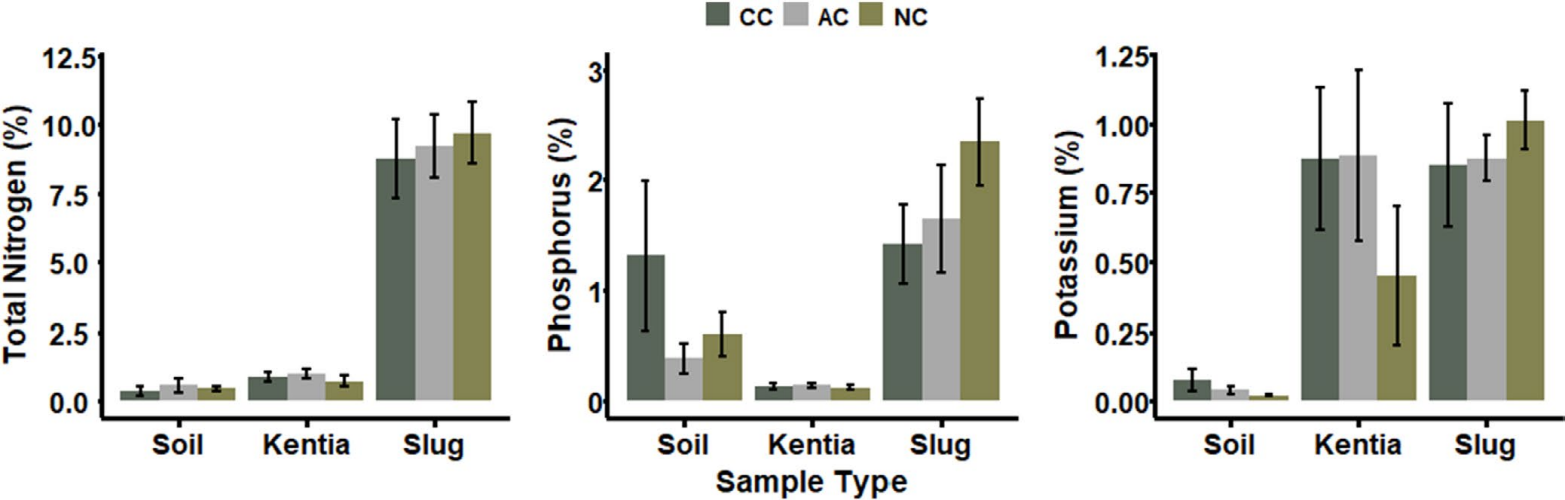
Concentrations (mean ± SD) of N, P and K (all %) in soil, kentia leaf and leopard slug samples from three sites on Lord Howe Island: an active Sable Shearwater colony (CC), an abandoned colony (AC; abandoned within the last 10–15 years) and an area that has never hosted a shearwater colony (NC). Bars are grouped by sample type and colored by site. See Table S4 for exact mean ± SD and test statistics.

Soil samples from the active Sable Shearwater colony were not significantly different from the abandoned colony and uncolonised site, at either of the sampling depths, based on total N (%; *H*_2_ = 5.15, *p* = 0.08). When *δ* ^15^N was used as a tracer for marine‐derived nitrogen, soils from the current colony (12.0‰ ± 0.9‰) were not significantly different from soils from the other two sites (without seabirds present; abandoned colony = 12.0‰ ± 0.5‰, uncolonised site = 11.7‰ ± 0.7‰; *H*_2_ = 0.44, *p* = 0.648; Figure 2, Table S4). *δ* ^13^C also did not differ among sites (*χ* ^2^ = 0.19, *p* = 0.91). However, soil from the colony was significantly higher in the other nutrients tested: P (1.31% ± 0.68%; *H*_2_ = 15.12, *p* < 0.001), K (0.07% ± 0.04%; *H*_2_ = 17.66, *p* < 0.001; Figure 3, Table S4).

**FIGURE 4:**
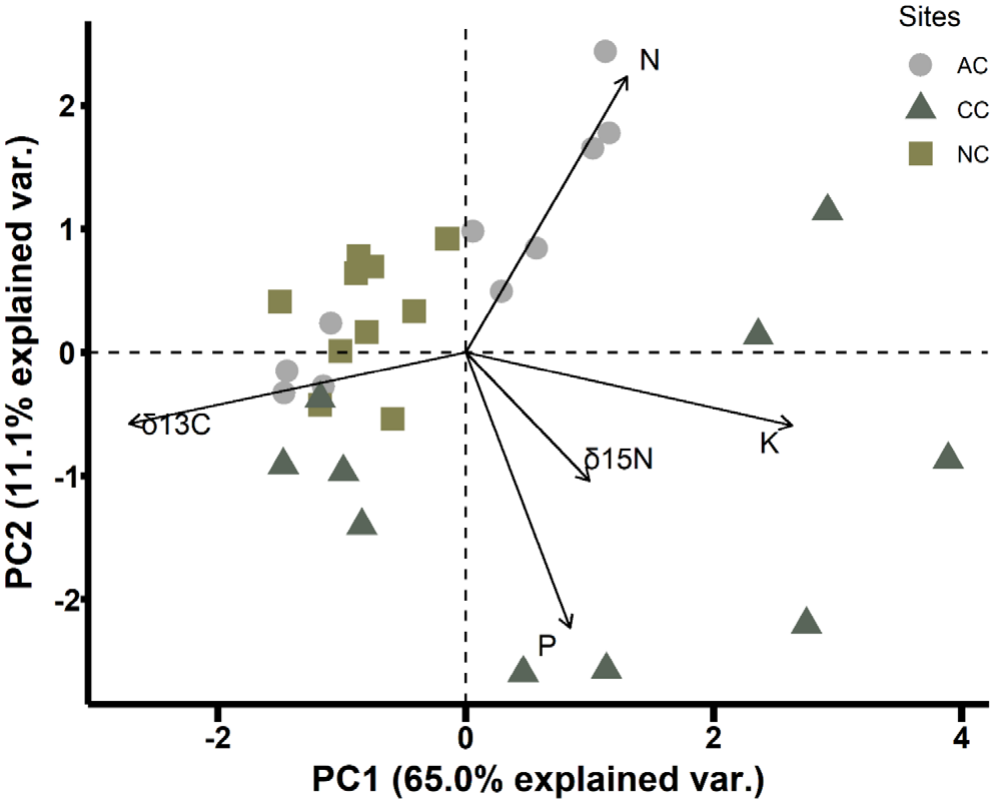
A principal component analysis (PCA) of nutrients and stable isotopes analysed in soil samples from a Sable Shearwater colony (CC; represented by triangles), an abandoned shearwater colony (AC; circles) and an area that has never had an active colony of shearwaters (NC; squares) on Lord Howe Island. Total N, P and K were reported as percentage (%), and *δ* ^15^N and *δ* ^13^C in permil (‰). Var., variance.

The first two principal components (PC1 and PC2) explained 76% of the total variance in nutrients and stable isotopes in soil samples across sites (Figure 4). The PCA showed that there was a clear grouping of soil samples from the uncolonised site and abandoned site, and the nutrients and stable isotopes in these were relatively similar, whereas there was more variation in nutrients and stable isotopes in soils from the currently occupied site (Figure 4). The first principal component (PC1) explained 65% of the variance in environmental variables among samples and had high loadings of K, N, *δ* ^15^N and P. The second (PC2) explained 11% of the variance and had negative loadings in *δ* ^13^C.

### 3.3 Nutrients and Stable Isotopes in Primary Producers and Consumers

The only significant difference in nutrients or stable isotopes in kentia palm leaves was for *δ* ^15^N (8.1‰ ± 0.8‰; *F*_2,12_ = 16.74, *p* < 0.001; Table S4). Leopard slugs from the current shearwater colony and the abandoned shearwater colony had significantly higher *δ* ^15^N values (13.4‰ ± 0.9‰ and 12.7‰ ± 0.8‰, respectively) compared to the uncolonised site (9.8‰ ± 0.7‰; *F*_2,28_ = 65.14, *p* < 0.001; Table S4). Interestingly, P was significantly greater in leopard slugs from the uncolonised site than the abandoned colony and currently occupied site (2.34% ± 0.39%; *F*_2,28_ = 15.99, *p* < 0.001) and the same pattern was observed for K (*F*_2,28_ = 3.98, *p* < 0.030; Table S4).

## 4 Discussion

### 4.1 Variation in Nutrients and Stable Isotopes in Soil and Primary Producers/Consumers

Our study provides evidence that there is a link between the Sable Shearwaters and the environments in which they breed on Lord Howe Island, however, the differences between the colony and the uncolonised site in our study were not as pronounced as has been reported for other systems (Grant et al. 2022). While some nutrients analysed in soil were significantly higher in the active shearwater colony (e.g., P and K), there were some nutrients that were expected to be higher yet were not, such as N. Much of the literature on the topic of seabirds as vectors of nutrients discuss how guano is rich in N and how this is commonly found in much higher concentrations in colony soils compared with control soils (De La Peña‐Lastra et al. 2020; Zwolicki et al. 2015), yet on Lord Howe Island, there was no difference in N among sites. Interestingly, the total N content (%) in Sable Shearwater guano was similar to other Procellariiformes compared in Grant et al. (2022; table 3), with a mean of 19.1% ± 3.5% across species, which tends to be higher compared to other seabird orders. This suggests that there are other factors that may be influencing nutrient concentrations, particularly N, in soils of shearwater colonies on Lord Howe Island.

On Lord Howe Island, a high proportion of N in excreted Sable Shearwater guano that is deposited on the surface of the colony is likely to be volatilised, given the subtropical climatic conditions of the island (see Section 2.1). Indeed, nitrogen addition through the deposition of guano does not necessarily equate to a greater concentration of N in soils as processes such as leaching, surface run‐off and volatilisation can occur (Hobara et al. 2005; Sobey and Kenworthy 1979). The N forms in guano are primarily uric acid (~80%), with small amounts of ammonia, nitrate and proteins (Mulder and Keall 2001; Szpak et al. 2012). Once guano has been deposited onto the colony surface, the uric acid is mineralised into ammonium and this can then be volatilised into the atmosphere as ammonia (Caut et al. 2012; Schmidt et al. 2004). The proportion of N that can be lost through volatilisation is impacted by climate and soil type (De La Peña‐Lastra 2021), with greater volatilisation occurring in warm and wet conditions (Schmidt et al. 2010). Furthermore, Riddick et al. (2012) estimated that when the average temperature is ≥ 19°C, all the N that is excreted is volatilised. However, volatilised N is not necessarily completely lost. Nesting habits of seabirds can influence ammonia emissions and relative N loss, with ammonia emissions from surface nesting seabirds greater than in burrowing seabirds (Hawke and Clark 2010a). Thus, as Sable Shearwaters are burrow nesters, ammonia emissions from guano that is deposited within the burrows (especially by chicks which spend 80–90 days within the burrow until they fledge) is likely to be reabsorbed within the soil of the burrow (Wilson et al. 2004).

While ammonia volatilisation is likely to be the main determining factor of N retention in soils, several other factors may play a minor role. Many previous studies on seabirds as vectors for nutrients have been based in comparatively low productivity and nutrient limited environments (e.g., little precipitation, few nutrient sources) like the Antarctic and Arctic (Abakumov 2018; Brimble et al. 2009) or in arid regions (Anderson and Polis 1999). In these areas, the influence of seabirds is often much more pronounced, and they can have a greater impact compared with areas that are less nutrient limited because soils in these regions can accumulate more N from guano (Gillham 1961; Grant et al. 2022). Lord Howe Island, in comparison, is a productive ecosystem surrounded by a productive marine environment. The island is bypassed by the East Australian Current and is surrounded by the world's southern‐most coral reef that is known for its high species richness and endemism (Harrison et al. 2011; Moriarty et al. 2023). The island receives a substantial amount of rainfall each year (see Section 2.1) which can also influence nutrient concentrations already in soils through leaching and runoff (Allaway and Ashford 1984; Smith 1978) (Figure S1), and these processes can be accelerated in soils with sparse plant cover (Bukancinski et al. 1994) or sandy soils (Bednik et al. 2020), like those in the shearwater colony. This means that guano, and the nutrients within, can be washed and diluted from the soil and the toxic effects of guano (i.e., guanotrophication, or seabird induced eutrophication) are not observed like they often are in arid environments (Gillham 1960).

Furthermore, the abandoned colony and the uncolonised site were adjacent to semi‐developed areas on the island. Due to the need for choosing sites with similar vegetation and soil structures as the Sable Shearwater colonies, we were limited to sites that were close to residential and hospitality (i.e., tourist accommodation) areas. It is possible that the nutrients in these sites were influenced by inputs by residents, including commercial fertilisers for gardens, burial of food scraps and septic tanks and this could influence the nutrient concentrations in soil, vegetation and soil invertebrates, particularly as these sites were downhill and within 30 m of inhabited areas. Furthermore, the introduction of livestock and cultivation in nearby areas may have influenced soil chemistry as well. Thus, in areas where the shearwaters breed on Lord Howe Island as well as other areas on the island (e.g., the abandoned colony and the uncolonised site) the input of N from a variety of sources (e.g., precipitation, artificial fertilisation, livestock) may have been equal to or greater than that of the input from shearwaters, meaning that the relative importance of the shearwaters in regulating the terrestrial environment of the island is less pronounced than if they were to inhabit a low productive environment (Markwell and Daugherty 2003) or one without human habitation.

While the concentration of N was not significantly different between soil samples across our three sampling sites, there were significantly higher concentrations of P and K in colony soils. In addition to guano containing considerable quantities of N, the other key nutrient found in guano is P and to a lesser extent, K (De La Peña‐Lastra 2021; Simpson et al. 2021). Soils from the active colony contained 2× more P and 3.5× more K than soils from the uncolonised area, indicating that the shearwater guano is likely responsible for fertilising soils in these areas. It is possible that the reason P and K are higher in soils from the current colony, while N is not, is due to N volatilisation, especially as all three nutrients can be influenced by leaching and run‐off (Sogn et al. 2018).

Globally, *δ* ^15^N values in terrestrial soils within seabird colonies can be as high as 20‰–45‰ (Hawke and Clark 2010b; Mizutani and Wada 1988), whereas soils not influenced by seabirds can have *δ* ^15^N values that are typically much lower (0‰–7‰; Mizutani et al. 1985, 1991). Our results for *δ* ^15^N values in soils from the currently occupied shearwater colony did not reach values as high as above, though they were higher than what is expected for soils not affected by seabirds (12.0‰ ± 0.9‰; Table S4). This indicates that soils from this site do contain marine‐derived content, with the Sable Shearwaters being the most likely source. However, mean *δ* ^15^N values of soil from the abandoned colony and the site that is uncolonised were not significantly lower, rather they were comparable to the current colony (12.0‰ ± 0.5‰ and 11.7‰ ± 0.7‰, respectively). While the results from the abandoned colony are unsurprising given that the effects of seabirds can be measured for many years after they have deserted a site (Boutin et al. 2011; Ellis et al. 2006) and long‐term effects can be greater in forests due to trees storing nutrients and continually supplying litter for the soil (Kameda et al. 2006), the results from the uncolonised site are unusual as this area has never hosted an active colony of Sable Shearwaters, or other seabird species, in recorded history. Paleolimnological analysis of soils and vegetation can provide data on fluctuations in seabird population size over centuries (Duda, Allen‐Mahé, et al. 2020; Duda, Glew, et al. 2020) with the influence of seabirds reliably measured in soils for up to 300–700 years after seabirds have departed (Markwell and Daugherty 2003). Given that Lord Howe Island has only been inhabited by humans for approximately 190 years, it is possible that this site had an active colony of seabirds prior to colonisation.

However, guano is not the only source of marine‐derived nutrients to terrestrial environments and thus not the only factor that may be responsible for increasing *δ* ^15^N in soil from the uncolonised site. Marine‐derived N can be carried through wind‐blown material which can lead to increased *δ* ^15^N with comparable values in local vegetation (Bokhorst et al. 2007). Sites can be enriched in *δ* ^15^N when they are in close proximity (< 1 km) to an area used by marine mammals (e.g., seal haul out) or seabird colony (Erskine et al. 1998). As Lord Howe Island hosts abundant marine life on the main island as well as the surrounding islets, wind‐blown material from these areas could be transported and deposited across the island. Furthermore, seabirds (of a variety of species) flying over the island could have deposited some guano within our uncolonised site which may also increase *δ* ^15^N (Bokhorst et al. 2007; González‐Bergonzoni et al. 2017; Schmidt et al. 2010).

There were no major differences in *δ* ^13^C between soil, primary producers or primary consumers across all three sites, which suggests that *δ* ^13^C may not be a good indicator for the influence of seabirds in the same way as *δ* ^15^N. Several other studies have come to similar conclusions (e.g., Cocks et al. 1998; Zwolicki et al. 2015) thus we suggest that future studies primarily focus on using N stable isotopes as a tracer for marine derived nutrients from seabirds.

Nutrients and stable isotopes in Kentia palm leaves did not differ among sites, except for in *δ* ^15^N. Palm leaves from the active colony had significantly higher *δ* ^15^N values than leaves sampled from the other two sites, which suggests that the kentia palms within the Sable Shearwater colony were incorporating more marine‐derived N. However, caution is needed when determining the relative contribution of sources of N in vegetation given that nitrogen form, concentration and cycling can have fractionation effects on *δ* ^15^N (Harding et al. 2004; Mizutani et al. 1985). Despite this, kentia palms within the Sable Shearwater colonies do appear to use N from the guano, but likely also incorporate N from a variety of other sources (Hawke and Newman 2007).

Nutrient concentrations and stable isotope values in leopard slugs were variable with some unexpected results (Table S4). Potassium and P were higher in slugs from the uncolonised site than in the abandoned colony and currently occupied colony. Interestingly, leopard slugs from the active colony and abandoned colony were enriched in *δ* ^15^N relative to the control site, which does suggest the slugs are consuming at least some material that contains marine‐derived nutrients. This aligns with data from other tropical islands where seabird‐derived nutrients have increased the nitrogen content of basal food sources, including gastropods and crabs (Appoo et al. 2024). However, the variable concentrations in other nutrients suggest leopard slugs are consuming a variety of food sources.

### 4.2 Correlation Between Sable Shearwaters and Kentia Palms

There is a visually apparent relationship between the presence of Sable Shearwaters and kentia palms within the colonies. While our study indicates that shearwater guano is rich in nutrients (Table 1), their co‐occurrence may also be driven by the physical disturbance caused by the shearwaters during their burrowing activity. Burrowing causes significant disturbance to vegetation, through damage to root structure (Bancroft, Roberts, and Garkaklis 2005). Kentia palm roots are adventitious and fibrous roots that grow out from the central trunk zone (Hodel et al. 2012), helping to stabilise plants in shifting environments (Steffens and Rasmussen 2015). The same can be said for banyan trees, which is one of the only other species present in the shearwater colonies (Sheringham et al. 2016). They also have adventitious root systems and aerial roots that form ‘root pillars’ to support the tree (Gonin et al. 2019). Given that the soil of shearwater colonies is sandy, and the colonies are a maze of burrows up to 3 m long, the adventitious root systems of kentia palms and banyan trees support these trees in the unstable soil. Thus, it is possible that the co‐location of shearwater colonies and kentia palms is due to the kentia palm roots creating a physical environment that is more conducive to shearwater burrowing rather than any nutrient benefit the kentia palms gain from growing in the proximity of shearwater colonies. Similar associations between seabirds and plants have previously been reported with the burrowing action of some seabirds known to result in distinct and less diverse vegetation communities (Bancroft, Roberts, and Garkaklis 2005).

### 4.3 Potential Outcomes of Sable Shearwater Population Decline

The Sable Shearwater population on Lord Howe Island has declined by as much as 50% since 2009 (Lavers et al. 2019). While a smaller population will mean a decrease in nutrients deposited by the shearwaters, it is unlikely that the kentia palms will suffer any negative consequences in the short term. Our results from the abandoned colony, in addition to results from a number of other studies (Boutin et al. 2011; Callaham et al. 2012; Markwell and Daugherty 2003), suggest the effects of shearwaters (i.e., the nutrients that have been deposited) will persist for an extended period even if the colony continues to decline. Thus, kentia palms will likely continue to thrive, especially given that they grow in other areas of the island where shearwaters are not present (see Section 2.1 for more detail) (Sheringham et al. 2016), which demonstrates that they are capable of surviving without the presence of shearwaters.

If the shearwater population continues to decline and the extent of their colony decreases, the vegetation structure in areas where shearwaters are no longer present may change. Vegetation assemblages in seabird colonies are often characterised by reduced species richness compared to uncolonised areas (Wait et al. 2005). This has also been observed in Procellariiform colonies elsewhere (Bancroft, Roberts, and Garkaklis 2005) and cormorants have been shown to be responsible for changing forest structure (Boutin et al. 2011). Vegetation thinning can be due to the physical activity of the birds (e.g., burrowing, trampling, using vegetation for nest construction; Sobey and Kenworthy 1979) which can also inhibit seedling growth and survival (Mulder and Keall 2001), or from chemical changes brought about by the addition of guano. For example, some plant species are not tolerant of high nutrient concentrations and may not survive in seabird colonies (Wait et al. 2005), and in extreme cases where excessive guano deposition leads to toxic effects, an elimination of all plants may occur (Gillham 1961). In addition to a decline in nutrient deposition, decreases in the Sable Shearwater population would also mean a reduction in physical disturbance. Together, this could lead to distinct changes in the habitat structure and species composition in areas where the shearwaters no longer breed. While plant species richness may increase (Bancroft, Roberts, and Garkaklis 2005), and species found elsewhere on the island (see Section 2.1) may shift into areas where the birds are absent, the loss of Sable Shearwaters would be significant for the island (i.e., as ecosystem engineers), and for the species.

## 5 Conclusion

Our study indicates that adult and chick Sable Shearwater guano have similar nutrient composition (Table 1); being rich in N, P and K, and is comparable to other Procellariiformes. This correlated with significantly higher concentrations of P and K in colony soils compared to soils from the control site and the abandoned colony. However, increased N concentrations were not reflected in colony soils in the same manner as P and K, which suggested that there are several factors that affect soil N concentrations on Lord Howe Island. Nitrogen volatilisation is responsible for a large component of N losses from the colony soils, with leaching and runoff possible factors as well, especially given the substantial amount of precipitation the island receives. Like N concentrations, *δ* ^15^N in soils were not significantly different across sites, and exhibited values that are reflective of a marine input. For the uncolonised site, this could point to the presence of a seabird colony prior to human habitation of the island.

While this study examined the chemical changes potentially brought about by the presence of Sable Shearwaters and did not critically explore the magnitude or influence of physical disturbance caused by the burrowing birds, this is a topic that would be interesting to explore in the future. Experimental manipulations could allow for distinguishing between chemical (i.e., guano) and physical (i.e., burrowing) effects. Our results show that Sable Shearwaters are chemical engineers, and their nesting habits confirm that they are also physical engineers, but further examination would provide additional clarification.

## Author Contributions

**Megan L. Grant:** data curation (equal), formal analysis (lead), investigation (equal), visualization (lead), writing – original draft (lead). **Suzie M. Reichman:** methodology (equal), writing – review and editing (equal). **Alexander L. Bond:** conceptualization (equal), formal analysis (equal), investigation (equal), supervision (equal), writing – review and editing (equal). **Jennifer L. Lavers:** conceptualization (equal), funding acquisition (lead), investigation (equal), methodology (equal), project administration (lead), supervision (equal), writing – review and editing (equal).

## Conflicts of Interest

The authors declare no conflicts of interest.

## Supporting information


Appendix S1.

## Data Availability

The data and code are available here https://doi.org/10.6084/m9.figshare.27985730.
