# Supplementary material for: Seabirds Enhance Primary Producer and Consumer Isotope Signals on a Sub‐Tropical Island
Source: Ecol Evol. 2025 Jul 27;15(7):e71636. doi: 10.1002/ece3.71636 (PMC12301070; doi:10.1002/ece3.71636)
Supplement: Supplementary file 1 — Appendix S1. [file ECE3-15-e71636-s001.docx]

**Seabirds enhance primary producer and consumer isotope signals on a sub-tropical island**

**Megan L. Grant, Suzie M. Reichman, Alexander L. Bond, Jennifer L. Lavers**

**Supplementary Information**

**TABLE S1.** Estimated nitrogen and phosphorus excreted (N_exc_ and P_exc_ in kilograms/year) by Sable Shearwater adults and chicks on Lord Howe Island during the breeding season. Calculations are based on a total population size of 22,645 breeding pairs (Lavers, Hutton, & Bond, 2019) and formulas adapted from Otero, De La Peña-Lastra, Pérez-Alberti, Ferreira, and Huerta-Diaz (2018).

| **Age** | **N_exc_** | **P_exc_** |
| --- | --- | --- |
| Adults | 2,603.7 | 433.9 |
| Chicks | 5.1 | 0.8 |

**TABLE S2** GPS coordinates for each sampling site on Lord Howe Island.

| **Site** | **Quadrat ID** | **Latitude (South)** | **Longitude (East)** |
| --- | --- | --- | --- |
| Current colony | 1 | 31°31'40.872" | 159°4'44.220" |
| Current colony | 2 | 31°31'40.332" | 159°4'44.436" |
| Current colony | 3 | 31°31'40.548" | 159°4'45.696" |
| Current colony | 4 | 31°31'42.240" | 159°4'44.004" |
| Current colony | 5 | 31°31'42.312" | 159°4'44.760" |
| Abandoned colony | 1 | 31°31'15.384" | 159°3'59.400" |
| Abandoned colony | 2 | 31°31'15.384" | 159°3'59.652" |
| Abandoned colony | 3 | 31°31'15.420" | 159°3'59.544" |
| Abandoned colony | 4 | 31°31'15.240" | 159°3'59.652" |
| Abandoned colony | 5 | 31°31'15.168" | 159°3'59.724" |
| Never colonised | 1 | 31°31'32.952" | 159°4'9.516" |
| Never colonised | 2 | 31°31'32.628" | 159°4'9.156" |
| Never colonised | 3 | 31°31'32.520" | 159°4'9.120" |
| Never colonised | 4 | 31°31'32.484" | 159°4'8.940" |
| Never colonised | 5 | 31°31'32.340" | 159°4'9.048" |

**TABLE S3** Certified concentrations/values and recovery of standard reference materials: CRM020 (Sandy Loam 2), NIST 1547 (peach leaves), and Dolt-5 (dogfish liver; all measured in mg/kg) analysed on ICP-MS; and Glycine, Glucose, and Collagen (*δ*^15^N and *δ*^13^C; ‰; total N% and C%) analysed on IRMS.

| **Reference material** | **Element** | **Certified concentration/Value** | **% Recovery** |
| --- | --- | --- | --- |
| CRM020 | K | 857 | 91 |
| NIST 1547 | P | 1371 | 102 |
|  | K | 24330 | 89 |
| Dolt-5 | P | 11500 | 91 |
|  | K | 14400 | 94 |
| Glycine | C | 32.8 | 97.8 |
|  | *δ*^13^C | -41.8 | 100.1 |
|  | N | 18.7 | 100.5 |
|  | *δ*^15^N | 2.0 | 103.4 |
| Glucose | C | 40.0 | 100.2 |
|  | *δ*^13^C | -10.5 | 100.0 |
| Collagen | C | 45.0 | 99.9 |
|  | *δ*^13^C | -21.5 | 100.0 |
|  | N | 16.3 | 99.8 |
|  | *δ*^15^N | 4.8 | 99.7 |

**TABLE S4** Differences in environmental variables between areas under varying degrees of seabird influence (an active Sable Shearwater colony, a colony that was abandoned within the last 20 years, and an area that has never been colonised by shearwaters) in soil, kentia palm leaves, and leopard slug *Limax maximus* samples. Means ± standard deviation (SD) are shown for every sample type and site. General linear models (test statistic = F) were run on normal data while Kruskal-Wallis tests were used for non-parametric data (test statistic = H, indicated by asterisk *). P values are shown in full unless p < 0.001. df = degrees of freedom. Superscript letters indicate significant differences, i.e., if sites for any given variable do not share the same letter, then they are significantly different from one another. Total N, P and K are measured as a percentage (%), δ15N and δ13C as per mil (‰).

|  |  | **Seabird influence (mean ± SD)** | | | **Test statistics** | | |
| --- | --- | --- | --- | --- | --- | --- | --- |
| **Sample** | **Variable** | **Active colony** | **Abandoned colony** | **Uncolonised site** | ***H*/F** | **df** | ***p* value** |
| Soil | N | 0.35 ± 0.16^a^ | 0.56 ± 0.28^a^ | 0.44 ± 0.10^a^ | 5.15* | 2 | 0.076 |
|  | P | 1.31 ± 0.68^b^ | 0.39 ± 0.13^a^ | 0.60 ± 0.19^ab^ | 15.12* | 2 | <0.001 |
|  | K | 0.07 ± 0.04^a^ | 0.04 ± 0.02^ab^ | 0.02 ± 0.01^b^ | 17.66* | 2 | <0.001 |
|  | *δ*^13^C | -13.3 ± 9.0^a^ | -10.3 ± 4.2^a^ | -8.6 ± 1.7^a^ | 0.19* | 2 | 0.908 |
|  | *δ*^15^N | 12.0 ± 0.9^a^ | 12.0 ± 0.5^a^ | 11.7 ± 0.7^a^ | 0.44 | 2,27 | 0.648 |
| Kentia leaves | N | 0.86 ± 0.18^a^ | 1.00 ± 0.17^a^ | 0.72 ± 0.22^a^ | 2.78 | 2,12 | 0.102 |
|  | P | 0.14 ± 0.03^a^ | 0.14 ± 0.03^a^ | 0.12 ± 0.02^a^ | 0.81 | 2,12 | 0.468 |
|  | K | 0.87 ± 0.26^a^ | 0.89 ± 0.31^a^ | 0.45 ± 0.25^a^ | 4.06 | 2,12 | 0.045 |
|  | *δ*^13^C | -29.3 ± 1.3^a^ | -30.5 ± 0.5^a^ | -29.1 ± 1.1^a^ | 2.68 | 2,12 | 0.109 |
|  | *δ*^15^N | 8.1 ± 0.8^b^ | 5.2 ± 0.8^a^ | 5.9 ± 0.8^a^ | 16.74 | 2,12 | <0.001 |
| Leopard slugs | N | 8.75 ± 1.45^a^ | 9.21 ± 1.17^a^ | 9.69 ± 1.11^a^ | 2.64* | 2 | 0.268 |
|  | P | 1.41 ± 0.36^a^ | 1.64 ± 0.49^a^ | 2.34 ± 0.39^b^ | 15.99 | 2,28 | <0.001 |
|  | K | 0.85 ± 0.22^a^ | 0.87 ± 0.08^ab^ | 1.01 ± 0.11^b^ | 3.98 | 2,28 | 0.03 |
|  | *δ*^13^C | -22.7 ± 0.5^b^ | -23.5 ± 0.9^a^ | -23.2 ± 0.5^ab^ | 4.31 | 2,28 | 0.023 |
|  | *δ*^15^N | 13.4 ± 0.9^b^ | 12.7 ± 0.8^b^ | 9.8 ± 0.7^a^ | 65.14 | 2,28 | <0.001 |

**
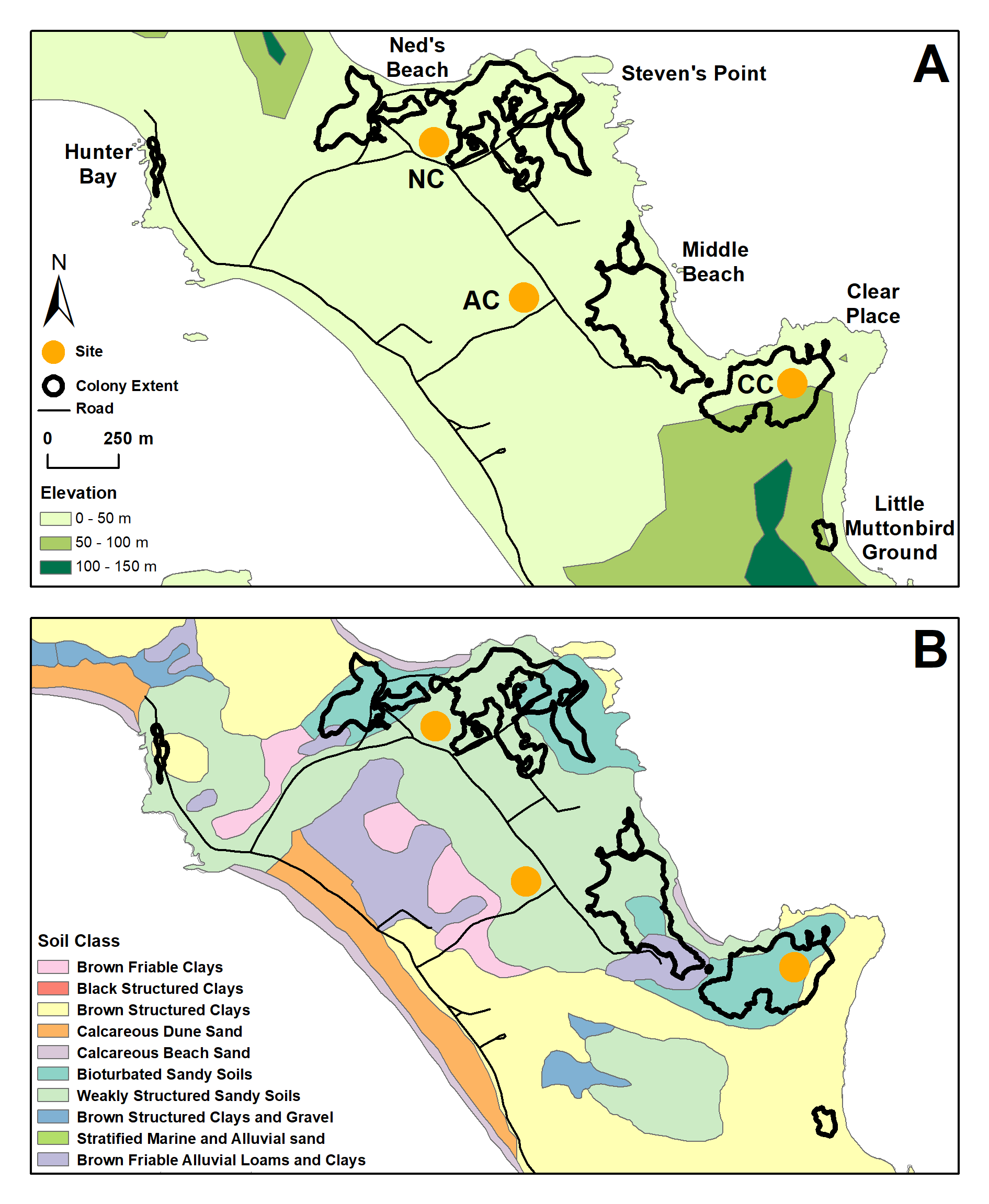
FIGURE S1** Elevation map (A) and soil class map (B) of the lowlands area of Lord Howe Island where the Sable Shearwaters breed (outlined in thick black lines: Hunter Bay, Ned’s Beach, Steven’s Point, Middle Beach, Clear Place, Little Muttonbird Ground). The three sampling sites are indicated by orange markers (AC = abandoned colony; NC = never colonised; CC = current colony).

**
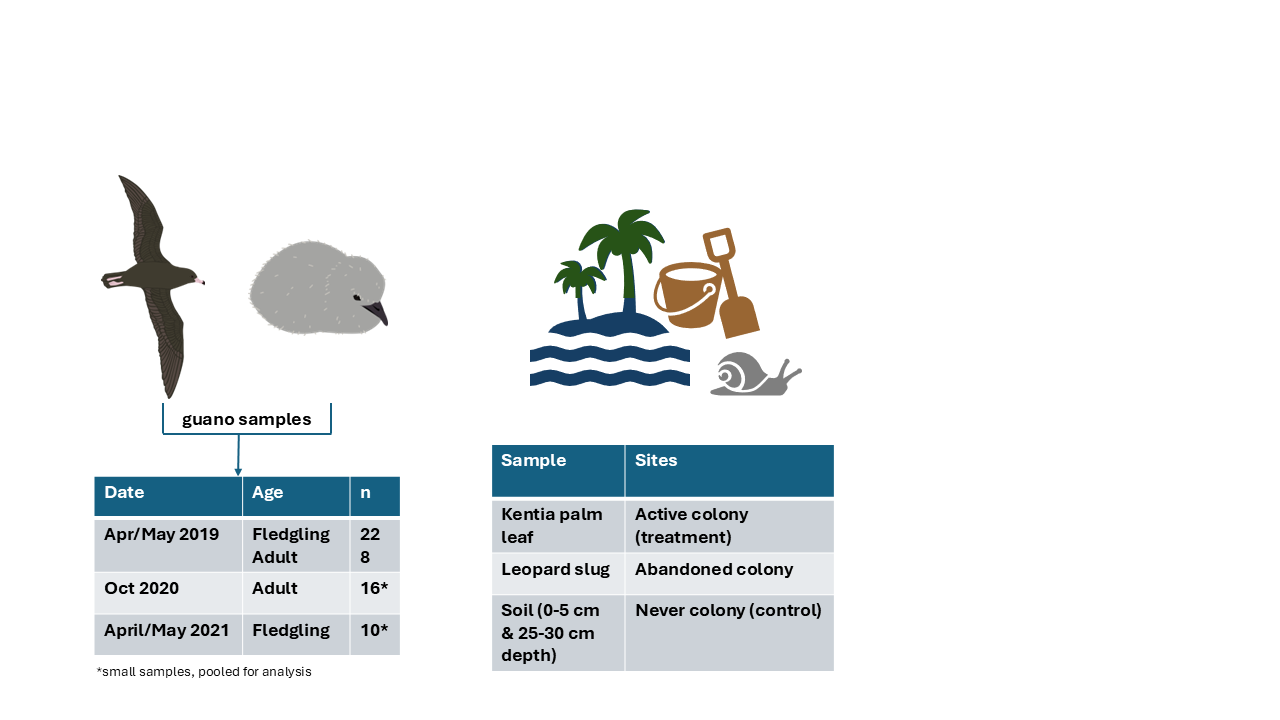
**

**FIGURE S2** Simplified study concept showing the date, number, type, and location of samples collected on Lord Howe Island.

**Supplementary references**

Lavers, J. L., Hutton, I., & Bond, A. L. (2019). Changes in technology and imperfect detection of nest contents impedes reliable estimates of population trends in burrowing seabirds. *Global Ecology and Conservation, 17*, e00579. doi:<https://doi.org/10.1016/j.gecco.2019.e00579>

Otero, X. L., De La Peña-Lastra, S., Pérez-Alberti, A., Ferreira, T. O., & Huerta-Diaz, M. A. (2018). Seabird colonies as important global drivers in the nitrogen and phosphorus cycles. *Nature Communications, 9*(1), 246. doi:<https://doi.org/10.1038/s41467-017-02446-8>
